# The “Regulator” Function of Viruses on Ecosystem Carbon Cycling in the Anthropocene

**DOI:** 10.3389/fpubh.2022.858615

**Published:** 2022-03-29

**Authors:** Yang Gao, Yao Lu, Jennifer A. J. Dungait, Jianbao Liu, Shunhe Lin, Junjie Jia, Guirui Yu

**Affiliations:** ^1^Key Laboratory of Ecosystem Network Observation and Modeling, Institute of Geographic Sciences and Natural Resources Research, Chinese Academy of Sciences, Beijing, China; ^2^College of Resources and Environment, University of Chinese Academy of Sciences, Beijing, China; ^3^Geography, College of Life and Environmental Science, University of Exeter, Exeter, United Kingdom; ^4^Carbon Management Centre, SRUC-Scotland's Rural College, Edinburgh, United Kingdom; ^5^Key Laboratory of Alpine Ecology, Institute of Tibetan Plateau Research, Chinese Academy of Sciences, Beijing, China; ^6^Chinese Academy of Sciences (CAS) Center for Excellence in Tibetan Plateau Earth Sciences, Chinese Academy of Sciences, Beijing, China; ^7^Department of Obstetrics and Gynecology, Fujian Maternity and Child Health Hospital, Fuzhou, China

**Keywords:** virus, carbon cycle, regulator, anthropogenic activity, climate change

## Abstract

Viruses act as “regulators” of the global carbon cycle because they impact the material cycles and energy flows of food webs and the microbial loop. The average contribution of viruses to the Earth ecosystem carbon cycle is 8.6‰, of which its contribution to marine ecosystems (1.4‰) is less than its contribution to terrestrial (6.7‰) and freshwater (17.8‰) ecosystems. Over the past 2,000 years, anthropogenic activities and climate change have gradually altered the regulatory role of viruses in ecosystem carbon cycling processes. This has been particularly conspicuous over the past 200 years due to rapid industrialization and attendant population growth. The progressive acceleration of the spread and reproduction of viruses may subsequently accelerate the global C cycle.

## Introduction

The scale of perturbation to Earth systems caused by human activity during the Holocene, and particularly over the last 2,000 years is now recognized as the Anthropocene epoch (1). Changes to Earth's ecosystems over millennia caused by human perturbation, including climate change, accelerating population growth and the globalization of trade and travel, have overridden biogeographic boundaries and allowed the rapid spread of viruses (2). This global phenomenon has drawn attention to the role of viruses in wider ecosystem functioning through their interactions with the global carbon cycle *via* the food web and the microbial loop in terrestrial and aquatic environments (3) that impose an indirect influence on climate change (4).

Disease-causing viruses diminish the fitness of their hosts, hinder development and reproduction, and may ultimately hasten their deaths (5, 6), driving the mineralization of organic carbon to inorganic carbon and its loss from food webs before it can flow to higher trophic levels (7). However, not all viruses are pathogens, and some are mutualistic, conferring benefits on hosts that include bacteria and fungi, plants, wasps and aphids, mice and humans (8). Indeed, current innovation in the treatment of cancers are developing the use of viruses to kill cancer cells selectively (9). Thus, viruses change the function of entire ecosystems by altering the abundances and community structures of organisms in food webs at every trophic level, from simple microorganisms (10, 11) to complex plants and animals (12).

**Figure 1:**
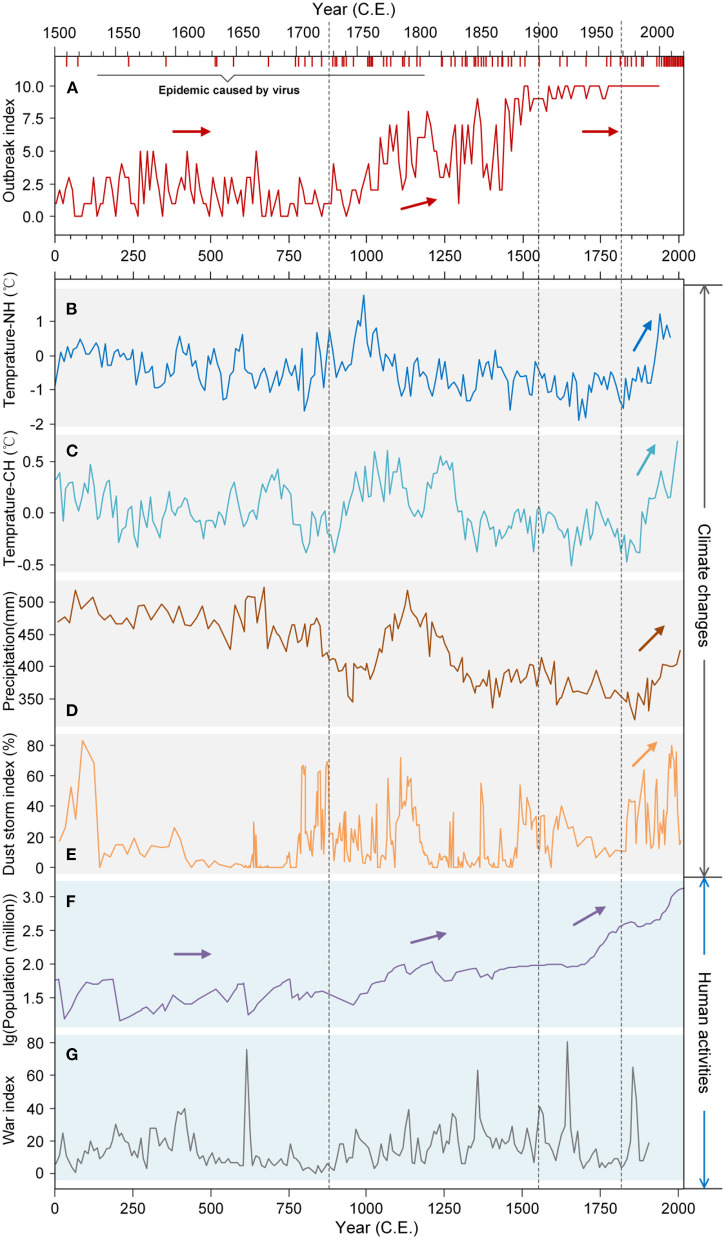
Global trends in climate change and anthropogenic activity and relationships with Chinese epidemic status over the last 2000 years. **(A)** The epidemic Outbreak Index of China [the number of years epidemics (bacteria, viruses and parasites) were recorded in China], and incidence of major global viral epidemic events. Each red vertical bar represents a viral epidemic event. See Supplementary Table S1 for detailed data information. **(B)** Temperature in the northern hemisphere (Temperature-NH); **(C)** temperature in China (Temperature-CH); **(D)** precipitation in the East Asian monsoon region; **(E)** dust storm index of North China; **(F)** population of China; **(G)** War Index, i.e., the total number of armed conflicts that occurred within China. Epidemic Outbreak Index values illustrating historical outbreak events can be roughly divided into three stages: the first stage (0 CE~1,000 CE) where values were all below 5; the second stage (1,000 CE~1,450 CE) of progressive increase in values from <5 toward 10; the third stage (1,450 CE~1,949 CE) where values plateau close to 10 (wherein epidemics occurred almost every year). CE, Chinese Common Era.

Natural fluctuations in climate have given way to human-induced global warming over the past 2,000 years, but most particularly since the beginning of the Chinese Common Era (CE) and European Industrial Revolution in the mid-18^th^ century and latterly the “Great Acceleration” of the Anthropocene since the 1950's. Progressive increases in average global temperatures have driven changes in rainfall patterns and caused more frequent and intense extreme weather events that have direct and indirect effects on viral epidemiology. Climate change affects the frequencies and durations of viral epidemics by altering the distribution, abundance and activity of hosts, changing resistance to infection, the physiology of host-virus interactions, the rate of virus evolution and host adaptation (13–16). Evidence suggests that global warming is leading to increased epidemics and, in turn, species extinctions. But the relationship between climate and epidemics may be different for different regions and different species (17). For example, recent evidence from European ice cores showed a strong relationship between unusual weather (low temperatures and high rainfall) and the severity of the Spanish Flu epidemic during the First World War (18). As another example, significant negative correlations are observed between temperature and precipitation and China's epidemic Outbreak Index (i.e., caused by bacteria, viruses or parasites); epidemics have tended to be relatively more frequent in China during colder and drier periods and relatively rarer during warmer and humid periods (Figure 1, Supplementary Table S1). Thus, it appears that climate change and viral epidemics are closely intertwined and interdependent with profound consequences for human, animal and environmental health, calling for the development of cross-disciplinary “One Health” strategies (19).

The current “black swan event” of COVID-19 has created an opportunity to observe how rapidly viral disease outbreaks can fracture ecosystem carbon flows by changing human behavior. Vastly reduced fossil fuel use during national lockdowns swiftly moved the global carbon balance toward a new state *via* regulatory feedback mechanisms (20) which may cause long-term and far-reaching changes to earth system interactions (21, 22). Thus, evidence is emerging that viruses can act as “regulators” of ecosystem carbon cycling through their effect on host (human) fitness and behavior, and that anthropogenic activity and climate change can alter viral epidemiology. However, the strength of the contributing factors to this exchange need to be identified to develop “One Health” solutions. Therefore, the objectives of this study were to (i) systematically clarify how viruses regulate carbon cycling processes, and (ii) reveal how anthropogenic activity and climate change influence the way that viruses regulate carbon cycling processes using published relevant data and findings. This study also proposes adaptive countermeasures to help combat any future influences of viruses on global C cycling processes.

## Methods

In order to systematically elucidate how virus regulate carbon cycling processes, we adopted the most commonly used calculation formula of contribution rate of C (CRC) in the world and the results of two published models to decompose the mechanism of virus in C cycle. We scraped data on virus abundance, as well as soil, ocean, and atmospheric C pools from different literature, and combined them into a mechanism diagram (**Figure 4**) to illustrate the impact scale of virus. To reveal the modulation of this process by anthropogenic activity and climate change, we use a China-wide dataset containing precipitation, dust storm index (DSI), temperature, population, and epidemic outbreak index.

### Modeling Viral Impacts on Ecosystem Carbon Cycles

This study applied the following formulae to estimate the CRC between viral lysing of bacteria and ecosystem DOC:


(1) $$\begin{array}{lll} \text{CRC} & = & \frac{\text{VLBC}}{\text{TCOE}} \end{array}$$

(2) $$\begin{array}{lll} \text{VLBC} & = & \text{FMVL}\times \text{BCP} \end{array}$$

(3) $$\begin{array}{lll} \text{MCP} & = & \text{PP}\times 20\times 10^{-9} \end{array}$$

(4) $$\begin{array}{lll} \text{FMVL} & = & \frac{\text{FVIC}}{\left[\gamma \text{ln}2\times \left(1-\varepsilon -\text{FVIC}\right)\right]} \end{array}$$

where TCOE is the total ecosystem DOC concentration (soil: mg C·kg^−1^/water: μg C·L^−1^); VLBC is the carbon released by viral lysing of bacteria (soil: mg C·kg^−1^/water: μg C·L^−1^); BCP is bacterial carbon production (soil: mg C·kg^−1^/water: μg C·L^−1^); BP is bacterial production (cell·L^−1^); FMVL is the fraction of mortality from viral lysis; FVIC is the frequency of visibly infected cells as seen under an electron microscope; γ is the ratio between the latent period and generation time; ε is the fraction of the latent period during which viral particles are not yet visible (23, 24). If γ = 1, ε = 0.186.

A steady-state model was used (shown in **Figure 4**) to determine the influence of virus under marine carbon cycling processes (25), which is a modification of the steady-state model developed by Jumars et al. (26) in that it allows for lysis of marine phytoplankton and marine bacterioplankton production. All values represent flux in photosynthetically fixed carbon (100%) and assume that all carbon in the pelagic zone eventually respires with negligible loss due to export. The data indicated that between 6 and 26% of the carbon fixed by primary producers enters the DOC pool *via* viral-induced lysis at different trophic levels (25).

This study applied the modified steady-state carbon flow model to determine a hypothetical aquatic microbial food web (27). The model showed that compared to a system devoid of virus, an otherwise identical food web with and without a viral component that is responsible for 50% of bacterial mortality and 7% of phytoplankton mortality underwent: (1) 33% more bacterial respiration and production; (2) 33% less bacterial grazing by protists; (3) 7% less microzooplankton production. The model confirmed the existence of a mechanism that showed that the viral lysis of phytoplankton would deprive the larger grazers and move material to smaller lifeforms.

### Data Sources

In this study, the data sets for precipitation, dust storm index (DSI), temperature, population, epidemic index and epidemic outbreak index data over the past 2,000 years were summarized from published data as well as published research. The 20-yr resolution precipitation data set shown in Figure 1 was based on pollen analysis from sediment cores in a reconstruction using the two-component weighted averaging partial least squares regression (WA-PLS) model (28). The dust storm index data set was reconstructed based on the coarse silt component (CSC) percentage in the sediment cores of Lake Gonghai (29). Northern Hemisphere temperature (Temperature-NH) data were reconstructed using the LOCal (LOC) method (30), and China temperature (Temperature-CH) data were reconstructed using principal component regression (PCR) and partial least squares (PLS) regression (31). Population, epidemic index and epidemic Outbreak Index data were extracted from regional publications and literature (32–34).

This study obtained total CO_2_ emissions (TCOE), frequency of visible infected cells (FVIC), fraction of mortality from viral lysis (FMVL), bacterial carbon production (BCP) and bacterial production (BP) data through analysis of relevant literature (Supplementary Table S2). Since bacteria comprise most soil microorganisms and there exists an integral relationship between soil microorganisms and viruses (35), soil BCP was substituted for soil microbial carbon production in this study. Moreover, Equation (2) assumes that the carbon content in each bacterial cell is constant (20 fg C·cell^−1^) (36). To date, no studies have been published on bacterial mortality caused by viral lysis in forest and desert soil. Therefore, we only estimated the CRC of wetland, cropland, pastureland and tundra ecosystem types. When the original data were presented in means or medians, the value was used directly; when the original data were a range, we used maximum and minimum values of the range for calculation.

Some data sets shown in **Figures 3**, **4** were extracted from published references (Supplementary Tables S3, S4). If the original data were a range, the median of the range was used. Floodplains and river reservoirs were regarded as lakes in this study. Data used in Supplementary Table S5 were extracted from the most recent global, regional and country-level estimates on cause-specific disability-adjusted life year (DALYs), years of life lost (YLL) and years lost due to disability (YLD) metrics for the years 2000, 2010, 2015 and 2016 (37).

## Viral Regulation of Ecosystem Carbon Cycling

Viruses regulate carbon cycling *via* their direct and indirect effects on the microbial loop and wider food web in terrestrial and aquatic ecosystems in three main ways.

**Figure 2:**
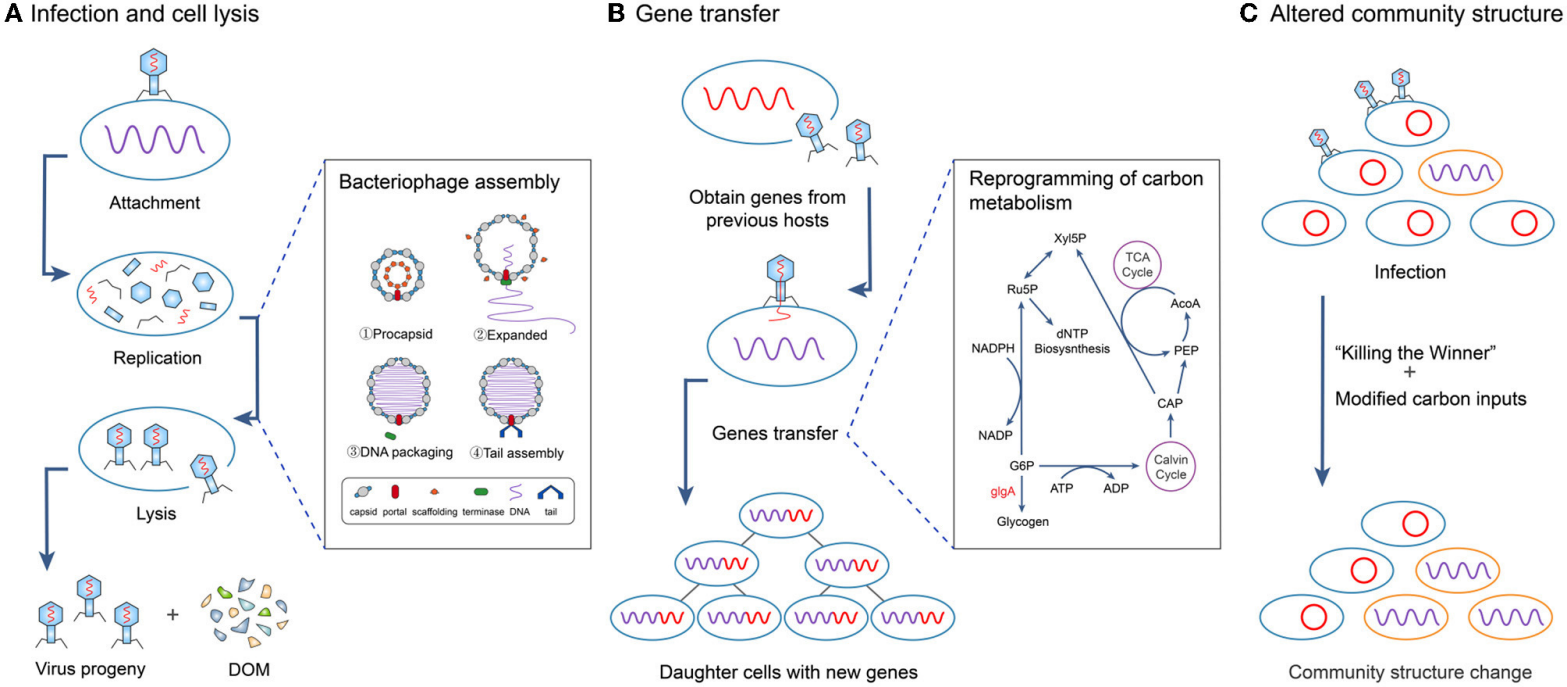
Three major mechanisms by which viruses affect microbial community structures and regulate carbon cycling. **(A)** Viruses infect microbial hosts and invade and destroy microbial cells (lysis) leading to the direct release of carbon in dissolved organic matter (DOM). **(B)** After virus infection, gene transfer from the virus (and/or previous host) previous host reprograms carbon metabolism. **(C)** Virus infection changes the magnitude of carbon inputs and changes microbial community structure, e.g., “Killing the Winner” mechanism.

(i) *Infection and cell lysis* Viruses (phages) accelerate the direct release of carbon from the microbial pool through microbial cell lysis (i.e., the “viral shunt”), especially bacteria in soils (35, 38–40) and plankton in aquatic systems (41–43) (Figure 2A).

(ii) *Gene transfer* Viruses indirectly regulate soil carbon cycling processes by affecting microbial host genes that encode for key biogeochemical functions, e.g., carbon metabolism and sporulation (44) through gene transfer (10, 11, 45), including the reprogramming of metabolic processes (becoming a “puppet master”) of the host cell (46), thereby regulating carbon (and nutrient) cycling (47–49) (Figure 2B). These genes include auxiliary metabolic genes (AMGs) that can regulate host photosynthesis (46, 50), carbon metabolism (51) and other such processes, which can alter the number, community structure and function of microorganisms (52).

(iii) *Altered community structure* Viruses alter the abundance, diversity and structure of microorganisms, including changing the dominance of microbial species [e.g.; “Killing the Winner” mechanism (53)] by modifying the magnitude of organic inputs. Viral infections of plants and animals in the wider food web may initially increase organic inputs due to increased mortality, but may ultimately reduce inputs by decreasing their abundance, e.g., viral infections of green plants can reduce rates of photosynthesis by up to 50% (54). Gene transfer can alter the availability of different organic substrates by mediating carbon source diversification processes (53, 55) which play an important role in maintaining species richness and the amount of available genomic information (52) (Figure 2C).

## Virus Distributions in Ecosystems

Viruses are extremely abundant infectious agents that are distributed throughout the biosphere (56), primarily in marine (55%) and freshwater (40%) ecosystems and to a much lesser extent in terrestrial ecosystems (<1%) (57).

**Figure 3:**
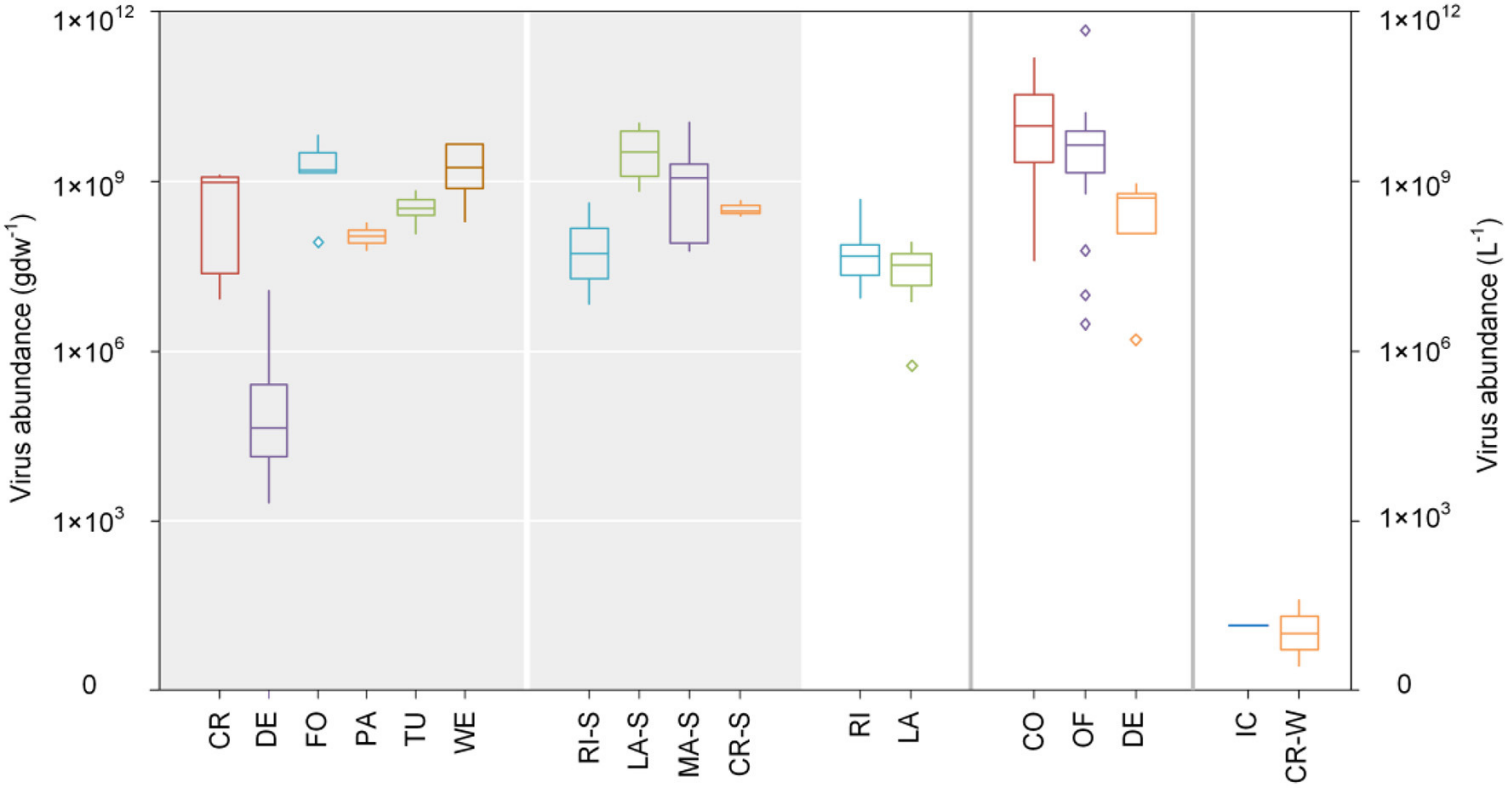
Virus abundance within different ecosystems. Grayed and transparent areas represent virus abundance values in solid and liquid matrices, respectively. All data in this figure were obtained through logarithms. CR, cropland; DE, Desert; FO, Forest; PA, Pasture; TU, Tundra; WE, Wetland; RI-S, River-Sediment; LA-S, Lake-Sediment; MA-S, Marine-Sediment; CR-S, Cryoconite holes-Sediment; RI, River; LA, Lake; CO, Coastal; OF, Offshore; DE, Deep sea; IC, Ice; CR-W, Cryoconite holes-Water. See Supplementary Table S3 for data sources.

In terrestrial systems, virions are easily adsorbed onto soil particles, and the degree of adsorption is commonly > 90% and reliant on soil properties including clay mineralogy, cation exchange capacity, soil organic matter and pH, as well as the type of virus (58). Thus, the migration rate of viruses in soil is very slow, which may explain why viruses have a weaker controlling effect on hosts in terrestrial ecosystems compared to freshwater and marine ecosystems (59, 60). Water availability and temperature control virus abundance in soils (40); desert soils have the poorest virus abundance (4.7 × 10^4^ gdw^−1^), while forests and wetlands have the largest (4.9 × 10^8^ gdw^−1^) (Figure 3).

The abundance of phytoplankton hosts of viruses in rivers and lakes is ~4.8 × 10^7^ L^−1^ and 3.5 × 10^7^ L^−1^ (Figure 3), respectively, which is frequently many times the magnitude of resident bacterial abundance (42). Virus abundance in river sediments is approximately 2.1 × 10^8^ gdw^−1^, which is less than in lake sediments (4.2 × 10^9^ gdw^−1^) (Figure 3). Virus abundance in rivers and lakes exhibit certain seasonal and spatial differences, wherein the peak of abundance generally occurs in summer and autumn (61). In wetland ecosystems, the average planktonic virus abundance is 2.7 × 10^10^ L^−1^, wherein corresponding abundances during the rainy and dry seasons are 4.4 × 10^10^ L^−1^ and 9.7 × 10^9^ L^−1^, respectively (62).

Virus abundance in marine ecosystems is > 10^30^ viruses, accounting for 89.7% of all viruses (63) and is ~10^8^~10^11^ L^−1^ in seawater. Compared to the seawater column, there are less viruses in marine sediments (1.1 × 10^9^ gdw^−1^) which is similar to the amount in lake sediments, and both hold more viruses than river sediments (Figure 3). There are more than 5,000 virus species in every 100 L of seawater and up to 1 million virus species per kilogram of marine sediment (45); consequently, viruses contribute ~94% of nucleic acid-containing particles in ocean water (10). Viruses exist in all marine environments, from shallow seas to deep oceans (64) and from low-latitudinal eutrophic regions to polar sea ice (48, 65) and their abundance is largest in the surface waters of tropical and subtropical oceans and smallest in polar regions. Virus abundance is least in the deep sea (5.2 × 10^8^ L^−1^) and mid-offshore surface waters (4.3 × 10^9^ L^−1^) and greatest in coastal waters (1.9 × 10^10^ L^−1^) (Figure 3).

## Viral Impacts on Ecosystem Carbon Cycles

**Figure 4:**
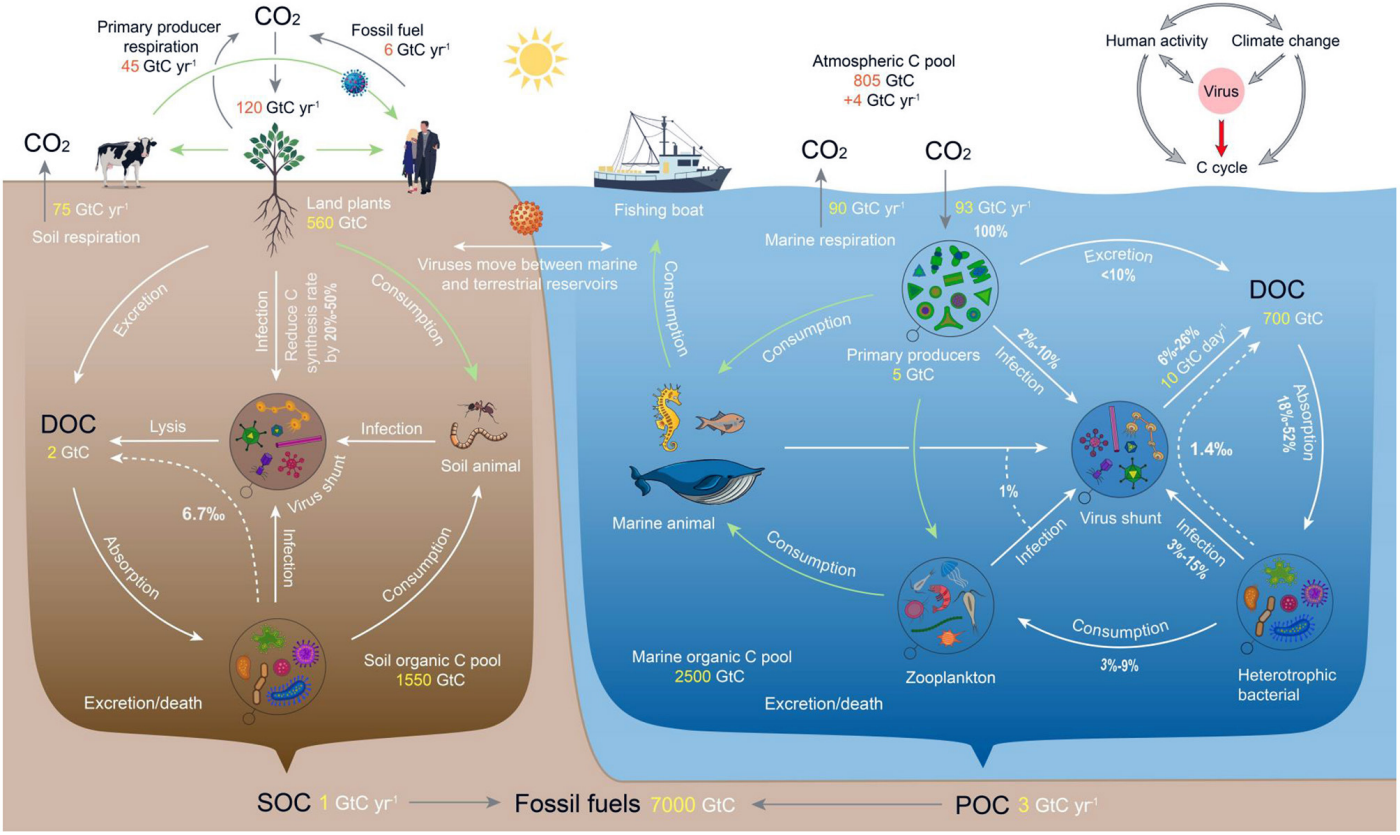
Viruses are a “regulator” of the global ecosystem C cycle network. The gray arrows in the upper right corner of the diagram represent influence and the red arrow represents regulation. The arrows show the roles that viruses play in the traditional food web, the “microbial loop” and the C cycle network of ecosystems. Light green arrows represent the traditional food web, white arrows represent the microbial loop, white dotted arrows represent the contribution rate of C produced by viral lysing of bacteria to the ecosystem DOC pool, and gray arrows represent the intersystem migration C process. Additionally, C reserves and the C exchange volume are indicated in orange or yellow font. The schematic diagram of the freshwater ecosystem was similar to that of the marine ecosystem and is not shown separately. The “microbial loop” is an important supplement to the classic food chain, wherein dissolved organic matter (DOM) is ingested by heterotrophic “planktonic” bacteria during secondary production. These bacteria are then consumed by protozoa, copepods and other organisms, and eventually returned to the classical food chain. DOM includes three categories according to biological availability: labile DOM (LDOM; ~26 Gt C), semi-labile DOM (SLDOM; ~50 Gt C) and recalcitrant DOM (RDOM; ~624 Gt C). All percentage values represent the flux of C fixed by primary producers (100%). See the Methods Section and Supplementary Table S4 for data sources.

By infecting and lysing microorganisms, viruses remove biomass from the main food chain and convert particulate organic carbon (POC) to dissolved organic carbon (DOC), forming a “viral shunt” pathway (Figure 4) which accelerates the flow of energy and carbon in the microbial loops of ecosystems (66–68). Most DOC circulates several times within the bacteria-virus-DOC cycle before being mineralized by the bacterial community, reducing the potential for transfer to higher trophic levels (69).

On land, DOC produced by viral lysis of bacteria contributes ~2.6–12.6‰ to the soil DOC pool (excluding forest and desert; to date, no studies have been published on bacterial mortality caused by viral lysis in forest and desert soils) (Figure 4, Supplementary Table S2). The scale at which viruses contribute a regulatory carbon cycle function differs between terrestrial ecosystems but is always important. Even in glacial ecosystems where temperature maxima are < 0.1°C, but that cover 15% of the landmass of the planet, viral activity persists and is relatively large in conditions that otherwise suppress most biological activity (70). In the four terrestrial ecosystems of Wetland, Cropland, Pasture and Tundra, viral lysis in tundra ecosystems contributed the most to soil DOC, producing carbon emissions of 927.1–4202.3 mg C·kg^−1^ and accounting for 2.9–22.2‰ of the total DOC pool, and least in wetland ecosystems, causing carbon emissions of 273.5–968.4 mg C·kg^−1^ and contributing 0.8–4.4‰ to the DOC pool (Supplementary Table S2). The reasons for the difference between terrestrial ecosystems are related to the potential for survival of viruses, and depends on the availability of appropriate hosts and, therefore, the factors controlling their community dynamics, e.g., water, temperature, carbon and nutrient availability (71), and management.

Viruses play an important role in the ecological regulation of lake carbon cycling processes, particularly in the flow and re-assimilation of organic carbon produced by bacterial lysis. In lake ecosystems, the mortality rate of bacteria caused by viral lysis ranges from 2.5 to 74.0%, which is larger than that caused by grazing by flagellates in certain lakes (72). The carbon emissions caused by this process range from 6.7 to 196.8 μg C·L^−1^, which account for 0.7–61.5‰ of the total DOC pool (Supplementary Table S2). In eutrophic lakes, ~29–79% of organic carbon may be reused and recycled within the bacterial-bacteriophage-DOC cycle (73). However, host mortality caused by viral lysis is larger in oligotrophic freshwater ecosystems and carbon release and recycling plays a critical role in microbial survival (74). Thus, in regions where the proportion of bacteria infected by virus is significantly larger, viruses may be the primary ecosystem regulators. In low-productivity freshwater ecosystems dominated by microorganisms (such as lakes in polar and high latitudinal regions), the microbial loop is the main flow pathway of energy and carbon (75, 76). For example, the carbon released by viral lysis is the main DOC source (60%) for lakes in Antarctica (77). Furthermore, the relative contribution of viral lysis to the DOC pool varies seasonally in polar and alpine regions where the rates in winter may be far greater (60%) compared to summer rates (<20%) (67). By comparison, in fluvial systems around one-third (33.6%, corresponding to 0.6 Pg C yr^−1^) of globally-respired carbon may pass through a viral loop (78). The proportion of bacterial mortality caused by viral lysis in rivers is 0.8–17.9%, emitting 2.1–47.6 μg C·L^−1^ and accounting for 0.4–8.4‰ of the total DOC pool.

In marine ecosystems, ~25% of ocean surface primary productivity passes through the “viral shunt” pathway (Figure 4), which results in the rapid circulation of DOC *via* an increase in community respiration and a 33% decrease in carbon transfer into higher trophic levels (79, 80). This mechanism promotes carbon use efficiency and maintains sufficient carbon in surface seawater and thus allows for greater oxidation (Figure 4), thereby regulating marine carbon cycles (81) within the largest C pool (82, 83). Here, phytoplankton, bacteria and other ocean microorganisms are the main contributors to DOC (84, 85) and between 6 and 26% of primary production enters the DOC pool *via* viral-induced lysis (Figure 4).

Viral lysis of bacteria has obvious spatial characteristics within different ocean environments. In offshore waters, viral lysis causes the release of 0.2–3.2 μg C·L^−1^, which accounts for 0.3–4.0‰ of the total organic carbon pool, while the release of carbon in coastal waters is 0.5–3.4 μg C·L^−1^ (Supplementary Table S2). Most DOC produced by viral-induced marine lysis is reincorporated by heterotrophic bacteria as POC *via* the microbial loop, with the remainder as DOC (8–42% in coastal waters and 6.8–25.0% in offshore waters). In deep sea sediments, both viral infections and lysis can lead to the death of > 80% of prokaryotes (or even 100% when water depth exceeds 1,000 m) (84), releasing a large amount of DOC into the deep sea, which significantly narrows the food chain and hastens organic carbon recycling. Overall, viruses boost primary production and sequestration in the deep ocean by helping to maintain nutrients in surface waters that are accessible to sunlight.

## Interactions Between Viruses, Anthropogenic Activity and Climate Change

**Figure 5:**
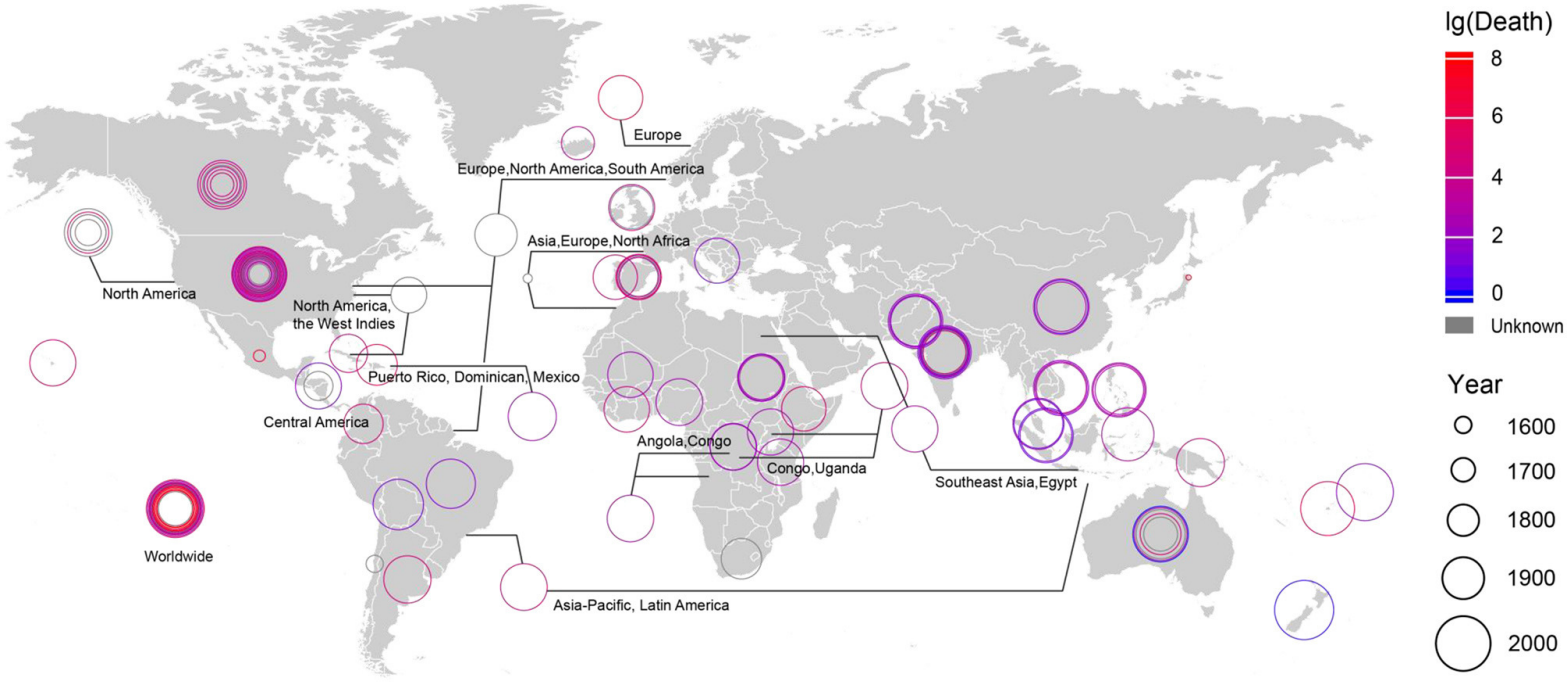
The outbreak time, location and death toll of all viral epidemics on record. See Supplementary Table S1 for detailed information.

The changing relationships between humans and their environment due to population increase and consumption of natural resources tend to closer proximity between humans, between humans and other species, and between humans and environmental virus pools, intensifying the potential for the spread of viral infection (Figure 5). From 2000 to 2016, the average human death rate caused by viruses was ~2.6 × 10^8^ people per year, accounting for 12.9% of the total global annual death rate (Supplementary Table S5).

Human activity, including urban expansion, biological resource utilization and viral disease control measures, changes the distribution and activity of viruses (14). Fluctuations or changes in the regulatory state of viruses may subsequently impact human welfare. For example, human viral disease, including HIV/AIDS, measles, encephalitis, hepatitis and lower and upper respiratory infections (37), are more frequent during periods of social unrest and armed conflict (Figure 1). Indirect effects of human activity on viruses include environmental pollutants, such as chemical fertilizers (86), pesticides (87) and heavy metals (88), that have diverse effects on virus dynamics (89). The expansion of crop irrigation and the international trade in plant products promote favorable conditions for widespread outbreaks and destructive viral epidemics (6).

Shifting global weather patterns caused by climate change affect the spread of viruses among people and vary between ecosystems and geographical regions (6), altering the frequency of severe epidemics (90). Increasing temperature, extreme precipitation events and droughts caused by climate change may facilitate the spread of viruses (91–94), including the release of viruses that have been stored for many millennia into the meltwaters of retreating glaciers (95). However, climate change may also reduce the incidence of viral disease; for example, an increase in temperature can enhance enzyme activities, promoting the degradation of viral capsid proteins (96).

The direct effects of the increasing incidence of human viral disease on the carbon cycle is becoming clear through our collective experience during the current global COVID-19 pandemic. Alteration of human behavior enforced by policy to reduce the risk of viral infection, such as self-isolation, reduced travel and employment deferment, have caused decreased global C emissions by −17 (ranging from −11 to −25) Mt CO_2_ d^−1^, a reduction of 27 to 14% compared to the 2019 mean emission level (20, 97–99). This immediate pandemic-driven response has unintentionally proven the potential of national policy to make a significant impact on the global carbon cycle. A managed reduction of greenhouse gas emissions to avoid global warming of 0.3°C by reducing 30–40 Gt fossil fuel CO_2_ emissions (22) appears to be achievable if long-term national socioeconomic polices are implemented.

Human well-being is threatened by insidious changes in viral epidemiology and climate change caused by anthropogenic activity. The global relationships between virus pandemics, global warming and human behavior is complex, but the overriding trend is toward the acceleration of the spread and reproduction of viruses, which may in turn accelerate the global carbon cycle. Overall, the prediction of virus regulation feedbacks in the Anthropocene must improve to provide theoretical and practical support that promotes the harmonious coexistence of humans and viruses as well as the stability and health of ecosystems globally.

## Unseen Impacts of COVID-19 on Global CO_2_ Emissions

**Figure 6:**
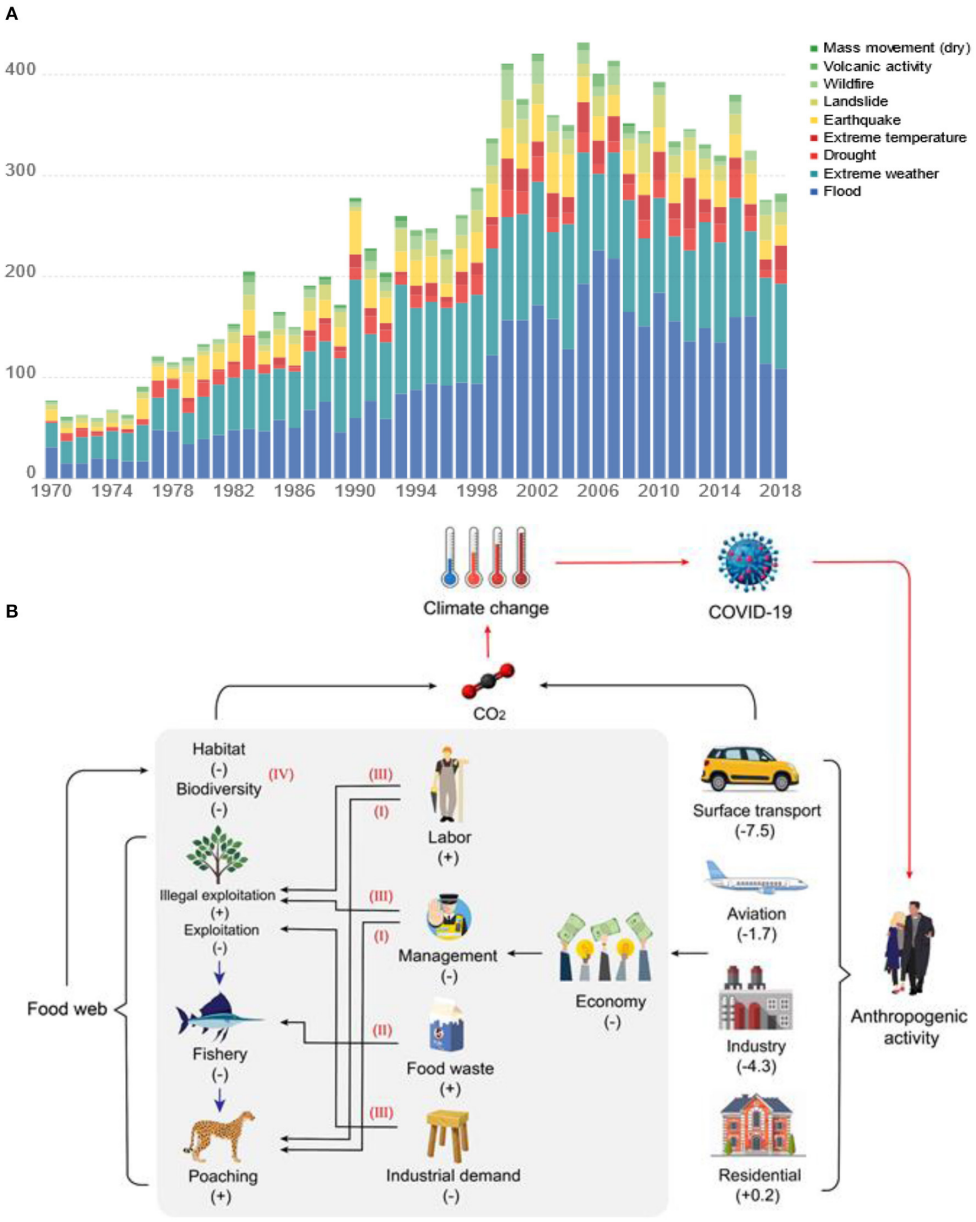
Global reported natural disasters by type for 1970–2019 (https://ourworldindata.org/natural-disasters) **(A)** and the impact of the COVID-19 pandemic on carbon emissions **(B)**. Proposed omissions in carbon emissions related to the food web are described within the area of gray shading. The red roman numerals I-IV correspond to estimation omissions described in the text. The red arrows outside the gray shaded area represent feedbacks and interactions within the virus-climate change-anthropogenic activity-carbon cycle continuum. “+” indicates that the component is promoted and “-” indicates that the component is weakened. The values in brackets are range in daily fossil CO_2_ emission on 7 April 2020 compared to mean daily 2019 levels ^5^, unit: MtCO_2_ day^−1^.

Historically, climate change and large-scale and sudden disasters have affected the survival and development of human societies, even triggering the rapid demise of great dynasties (100). Progressive growth of the global population enabled by technological progress has deepened the penetration of human activities into “ecosystem Earth” (101). Emerging interrelationships between climate change, anthropogenic activity and material cycles have been established. The intensification of globalization and global climate change since the beginning of the 20th century have co-occurred with the increased frequency of ecological catastrophes including human- and animal-borne diseases, biosecurity threats and super pests, and “natural disasters” such as extreme temperatures, large-scale forest fires, floods and droughts (Figure 6). Pressure on natural systems to meet increasing human demand for food and other animal products is driving increased emissions of CO_2_ [currently 26% (102)]. Observed changes in the relationship between people and the wider food web during the COVID-19 pandemic presents opportunities to alter future trajectories of CO_2_ emission from this source.

During 2020, restrictive policies on human activity imposed in response to the spread of COVID-19 in many countries and states across the world have seriously impacted the performance of global markets, leading to building pressure within national governments to release restrictions on human activity to support economic recovery. However, a beneficial by-product of the restrictive policies is a significant reduction in short-term carbon emissions caused by the change in human behavior (20, 22, 98), leading to calls for governments to use this opportunity to formulate and implement Green Economic Recovery policies with the potential to reduce global warming to rates within planetary boundaries (22). Climate-related disasters this year (such as storms in Fiji, flooding in the middle and lower reaches of the Yangtze River in China, droughts in southern African, and bushfires in Australia and California in the United States) and the epidemic are intertwined (99). Poor human health, caused by exposure to the consequences of climate-driven disasters and other human-driven stressors of ecosystems, promotes susceptibility to COVID-19 infection; for example, lung disease due to increases in PM_2.5_ caused by industrial air pollution (103) and wildfires. Balancing appropriate responses to these interdependent phenomena poses a tremendous policy challenge because of the growing recognition of feedbacks and interactions between the spread and severity of the virus, anthropogenic activity, the carbon cycle and climate change.

Global “black swan” events such as infectious disease outbreaks can alter carbon emissions over the short-term and may potentially affect the carbon balance of the Earth's ecosystems over the long-term. Viruses play key roles in regulating ecosystem carbon cycling processes by impacting material cycles and energy flows in the food web and the microbial loop that regulates CO_2_ emissions from organic matter decomposition, under the influence of anthropogenic activity and climate change. Thus, sudden and large-scale viral outbreaks function as “regulators” of the global carbon cycle with the potential to rapidly sever the world's ecosystem carbon balance over a short timeframe (104). We are actively witnessing the importance of the COVID-19 pandemic as a factor in the reduction of anthropogenic-driven short-term carbon emissions, but are unable to yet comprehend the potentially far-reaching and longer-term impacts on carbon emissions from the entire food web, a factor which has not been taken into account in recent carbon emission estimation studies. Therefore, we propose that major estimation omissions have been made to actual carbon emission changes and the climate effects that these changes engender, that are created by human responses to the COVID-19 pandemic.

We propose that the reduction in emissions could be moderated *via* direct and indirect impacts on the economic activities of human society, particularly the consumption of animals as food or for leisure activity (Figure 1B). Potential unaccounted estimation omissions during the COVID-19 pandemic include:

(I) A halt in tourism and the withdrawal of labor from nature reserves have led to an increase in wildlife poaching [for example, recent rhino horn poaching incidents in India (105) and raptors and fish in Europe (106)] and financial crises in zoos and wildlife rehabilitation centers threaten the survival of species important for ecotourism, including orangutans in Borneo (107).

(II) Shrinking fresh food markets selling farmed and wild animal products in some regions including China and Africa (108), have led to a decrease in the legal capture of wild aquatic and terrestrial animals, with the fishing industry most affected (109); whilst direct sales of fresh produce from farms has increased as western consumers seek local and traceable food options (110). Globally, the pandemic has disrupted the food supply chain system. Disruptions in food markets and workforces are causing a doubling of people facing severe hunger and huge amounts of land, fertilizer, energy and water being wasted. Among them, food waste has increased from about 8% of global anthropogenic greenhouse gas emissions to a larger proportion. In India, migrant workers are confined to their home villages, leaving fresh fruit unpicked and rotting in the fields. In the United States, the embodied carbon footprint of livestock and dairy losses have reached at least 7.1 MtCO_2_e. In the EU, the carbon footprint of potato waste (one of the lowest carbon footprint foods) comes to 0.5 MtCO_2_e (111).

(III) The global economic slowdown has decreased demand for industrially-produced commodities, thereby reducing direct environmental pressure (112); however, the decline in centralized management of protected areas may lead to higher rates of unlawful resource exploitation, such as illegal logging that causes the emission of previously sequestered carbon from standing biomass and degraded soils (113).

(IV) In economically deprived regions, spikes in unemployment and the loss of family income have increased the dependency on local natural resources for wild sources of food and fuel, and the increased exploitation of marginal lands for agriculture, increasing risks to ecosystem integrity associated with habitat and biodiversity loss (112, 114).

The prolonged economic downturn caused by the COVID-19 pandemic and resulting series of policy decisions during recovery may have a more profound and lasting impact on carbon emissions (21). We identified two dominant factors linked to changes in global carbon emissions caused by the COVID-19 pandemic, (1) the widely acknowledged reduction in carbon emissions through the sudden decline in fossil fuel use caused by a decrease in anthropogenic activity, and (2) the less well-documented change in carbon emission rates caused by the cumulative impact of altered human behavior propagating through the food web. We hypothesize that the net effect of these two factors on the environment is comparable to the effect of human population decrease because the degree of human intervention in the ecological environment during the viral outbreak is reduced, which is similar to the impact of population decline. In other words, a proportion of the reduction in overall carbon emissions is due to Earth ecosystem compensation and feedback mechanisms, resulting in a longer-term slowdown in carbon emissions than estimated through traditional methods. However, as we have described, the balance between promoting or reducing CO_2_ emissions for the long term depends on the policy-driven encouragement of altered patterns of human consumption that reduce pressure on the natural environment *via* the food web.

## Conclusion

Human well-being is threatened by insidious changes in viral epidemiology and climate change caused by anthropogenic activity. The global relationships between virus pandemics, global warming and human behavior is complex, but the overriding trend is toward the acceleration of the spread and reproduction of viruses, which may in turn accelerate the global carbon cycle. Overall, the prediction of virus regulation feedbacks in the Anthropocene must improve to provide theoretical and practical support that promotes the harmonious coexistence of humans and viruses as well as the stability and health of ecosystems globally.

The maintenance of Earth ecosystem integrity is crucial for the future sustainability of human society. COVID-19 has provided us with insight into the capability of people to effect change collaboratively in the face of a common threat. Post-pandemic, due to lags in feedback systems, the indirect effects of a short-term reduction in anthropogenic activities will gradually and distinctly manifest after lockdown restrictions are lifted, potentially altering the status of the carbon cycle balance of Earth's ecosystems for the long-term. Therefore, it is essential to secure a full comprehension of the role that virus plays in global carbon cycling to aid efforts to obtain more accurate measurements of actual carbon emissions.

During the formulation of COVID-19 economic recovery policies, policymakers must look beyond direct changes to carbon emissions to the role and contribution of indirect changes in carbon emissions. Critically, there is an urgent need for research to establish how changes in anthropogenic activities resonate through the food web and their consequent expression as indirect contributions to carbon emissions. This will allow for a more comprehensive and accurate platform from which to judge overall ecosystem carbon emissions. Globalization, urbanization and climate change are driving increases in human connectivity making future global viral epidemics inevitable. In response, we must attend to issues related to maintaining ecosystem integrity to inform appropriate policy responses through a detailed understanding of impacts and feedbacks within the climate change-anthropogenic activity-carbon cycle continuum.

## Data Availability Statement

The original contributions presented in the study are included in the article/Supplementary Material, further inquiries can be directed to the corresponding authors.

## Author Contributions

YG and GY: conceptualization. JJ, SL, and JL: resource and data curation. YL, JD, and YL: writing-original draft. YG and JD: writing-review and editing. All authors contributed to the article and approved the submitted version.

## Funding

This study was financially supported by the National Nature Science Foundation of China (No. 31988102, 41922003, and 42141015).

## Conflict of Interest

The authors declare that the research was conducted in the absence of any commercial or financial relationships that could be construed as a potential conflict of interest.

## Publisher's Note

All claims expressed in this article are solely those of the authors and do not necessarily represent those of their affiliated organizations, or those of the publisher, the editors and the reviewers. Any product that may be evaluated in this article, or claim that may be made by its manufacturer, is not guaranteed or endorsed by the publisher.
