# Supplementary material for: The “Regulator” Function of Viruses on Ecosystem Carbon Cycling in the Anthropocene
Source: Front Public Health. 2022 Mar 29;10:858615. doi: 10.3389/fpubh.2022.858615 (PMC9001988; doi:10.3389/fpubh.2022.858615)
Supplement: Supplementary file 1 [file Data_Sheet_1.pdf]

Table S1 List of major viral epidemics on record

Event	Date	Location	Disease	Death toll
735–737 Japanese Smallpox epidemic	735–737	Japan	Smallpox	2 million (approx. 1⁄3 of Japanese population)
1510 influenza pandemic	1510	Asia, North Africa, Europe	Influenza	Unknown, around 1% of those infected
1520 Mexico Smallpox epidemic	1519–1520	Mexico	Smallpox	5–8 million (40% of population)
1561 Chile Smallpox epidemic	1561–1562	Chile	Smallpox	Unknown (20-25% of native population)
1592–1596 Seneca nation measles epidemic	1592–1596	Seneca nation, North America	Measles	Unknown
Massachusetts Smallpox epidemic	1633–1634	Massachusetts Bay Colony, Thirteen Colonies	Smallpox	1,000
1634–1640 Wyandot people epidemic of infections	1634–1640	Wyandot people, North America	Smallpox and Influenza	15,000–25,000
1648 Central America yellow fever epidemic	1648	Central America	Yellow fever	Unknown

1677–1678 Boston Smallpox epidemic	1677–1678	Massachusetts Bay Colony, United States	Smallpox	750–1000
1699 Charleston and Philadelphia yellow fever epidemic	1699	Charleston and Philadelphia, United States	Yellow fever	520 (300 in Charleston, 220 in Philadelphia)
1702 New York City yellow fever epidemic	1702	New York City, United States	Yellow fever	500
1702–1703 St. Lawrence Valley Smallpox epidemic	1702–1703	New France, Canada	Smallpox	1,300
1707–1709 Iceland Smallpox epidemic	1707–1709	Iceland	Smallpox	18,000+ (36% of population)
1713–1715 North America measles epidemic	1713–1715	Thirteen Colonies and New France, Canada	Measles	Unknown
1721 Boston Smallpox outbreak	1721–1722	Massachusetts Bay Colony	Smallpox	844
1730 Cádiz yellow fever epidemic	1730	Cádiz, Spain	Yellow fever	2,200
1732–1733 Thirteen Colonies influenza epidemic	1732–1733	Thirteen Colonies	Influenza	Unknown
1733 New France Smallpox epidemic	1733	New France, Canada	Smallpox	Unknown

1738–1739 North Carolina Smallpox epidemic	1738–1739	Province of Carolina, Thirteen Colonies	Smallpox	7,700–11,700
1739–1740 Thirteen Colonies measles epidemic	1739–1740	Thirteen Colonies	Measles	Unknown
1741 Cartagena yellow fever epidemic	1741	Cartagena, Colombia	Yellow fever	20,000
1747 Thirteen Colonies measles outbreak	1747	Thirteen Colonies	Measles	Unknown
1759 North America measles outbreak	1759	North America	Measle	Unknown
1760 Charleston Smallpox epidemic	1760	Charleston, United States	Smallpox	730–940
1761 North America and West Indies influenza epidemic	1761	North America, West Indies	Influenza	Unknown
1762 Havana yellow fever epidemic	1762	Havana, Cuba	Yellow fever	8,000
1763 Pittsburgh area Smallpox outbreak	1763	North America, present-day Pittsburgh area	Smallpox	Unknown
1772 North America measles	1772	North America	Measles	Unknown

epidemic				
1775–1776 England influenza outbreak	1775–1776	England	Influenza	Unknown
1775–1782 North American Smallpox epidemic	1775–1782	Pacific Northwest, United States	Smallpox	11,000+
1778 Spain dengue fever outbreak	1778	Spain	Dengue fever	Unknown
1788 Pueblo Indians Smallpox epidemic	1788	Pueblo Indians, Southwestern United States	Smallpox	Unknown
1788 United States measles epidemic	1788	United States	Measles	Unknown
1789–1790 New South Wales Smallpox epidemic	1789–1790	New South Wales, Australia	Smallpox	Unknown (50–70% of native population)
1793 United States influenza and *** epidemic	1793	United States	Influenza	Unknown
1793 Philadelphia yellow fever epidemic	1793	Philadelphia, United States	Yellow fever	5,000+
1800–1803 Spain yellow fever epidemic	1800–1803	Spain	Yellow fever	60,000+

1802–1803 Saint-Domingue yellow fever epidemic	1802–1803	Saint-Domingue	Yellow fever	29,000–55,000
1820 Savannah yellow fever epidemic	1820	Savannah, Georgia, United States	Yellow fever	700
1821 Barcelona yellow fever epidemic	1821	Barcelona, Spain	Yellow fever	5,000–20,000
1828–1829 New South Wales Smallpox epidemic	1828–1829	New South Wales, Australia	Smallpox	19,000
1831–1834 Plains Indians Smallpox epidemic	1831–1834	Plains Indians	Smallpox	Unknown
1837 Great Plains Smallpox epidemic	1837–1838	Great Plains, United States and Canada	Smallpox	17,000+
1840 South Africa Smallpox epidemic	1840	South Africa	Smallpox	Unknown
1841 Southern United States yellow fever epidemic	1841	Southern United States (especially Louisiana and Florida)	Yellow fever	3,498
1847 Southern United States yellow	1847	Southern United States (especially	Yellow fever	3,400

fever epidemic		New Orleans)		
1847–1848 influenza epidemic	1847–1848	Worldwide	Influenza	Unknown
1848–1849 Hawaii epidemic of infections	1848–1849	Hawaiian Kingdom	Measles, whooping cough, dysentery and Influenza	10,000
1850–1851 North America influenza epidemic	1850–1851	North America	Influenza	Unknown
1853 New Orleans yellow fever epidemic	1853	New Orleans, United States	Yellow fever	7,970
1855 Norfolk yellow fever epidemic	1855	Norfolk and Portsmouth, England	Yellow fever	3,000 (2,000 in Norfolk, 1,000 in Portsmouth)
1857 Lisbon yellow fever epidemic	1857	Lisbon, Portugal	Yellow fever	6,000
1857 Victoria Smallpox epidemic	1857	Victoria, Australia	Smallpox	Unknown
1857–1859 Europe and the Americas influenza epidemic	1857–1859	Europe, North America, South America	Influenza	Unknown
1862–1863 British Columbia Smallpox epidemic	1862–1863	British Columbia, Canada	Smallpox	32,000
1867 Sydney measles epidemic	1867	Sydney, Australia	Measles	748

1871 Buenos Aires yellow fever epidemic	1871	Buenos Aires, Argentina	Yellow fever	13,500–26,200
1870–1875 Europe Smallpox epidemic	1870–1875	Europe	Smallpox	500,000
1875 Fiji measles outbreak	1875	Fiji	Measles	40,000
1878 New Orleans yellow fever epidemic	1878	New Orleans, United States	Yellow fever	4,046
1878 Mississippi Valley yellow fever epidemic	1878	Mississippi Valley, United States	Yellow fever	13,000
1885 Montreal Smallpox epidemic	1885	Montreal, Canada	Smallpox	3,164
1889–1890 flu pandemic	1889–1890	Worldwide	Influenza or HCoV-OC43[142][143] (disputed)	1 million
Papua New Guinea kuru epidemic	1901–2009	Papua New Guinea	Kuru	2,700–3,000+
1918 influenza pandemic ('Spanish flu')	1918–1920	Worldwide	Influenza: A virus subtype H1N1	50 million+ (17–100 million) - (3-5% world's population)
1924–1925 Minnesota Smallpox epidemic	1924–1925	Minnesota, United States	Smallpox	500

1940 Sudan yellow fever epidemic	1940	Sudan	Yellow fever	1,627
1957–1958 influenza pandemic ('Asian flu')	1957–1958	Worldwide	Influenza:A virus subtype H2N2	1–4 million
1960–1962 Ethiopia yellow fever epidemic	1960–1962	Ethiopia	Yellow fever	30,000
Hong Kong flu	1968–1970	Worldwide	Influenza: A virus subtype H3N2	1–4 million
1972 Yugoslav Smallpox outbreak	1972	Yugoslavia	Smallpox	35
London flu	1972–1973	United States	Influenza: A virus subtype H3N2	1,027
1974 Smallpox epidemic of India	1974	India	Smallpox	15,000
Soviet flu	1977–78	Worldwide	Influenza: A virus subtype H1N1	10,000–30,000
HIV/AIDS pandemic	1981–present (data as of 2018[update])	Worldwide	HIV/AIDS	32 million+ (23.6–43.8 million)
1986 Oju yellow fever epidemic	1986	Oju, Nigeria	Yellow fever	5,600+

1987 Mali yellow fever epidemic	1987	Mali	Yellow fever	145
1998–99 Malaysia Nipah virus outbreak	1998–1999	Malaysia	Nipah virus infection	105
2000 Central America dengue epidemic	2000	Central America	Dengue fever	40+
2002–04 SARS outbreak	2002–2004	Worldwide	Severe acute respiratory syndrome (SARS)	774
Avian influenza outbreaks in the 2000s	2003–2019	Southeast Asia and Egypt	Influenza: A virus subtype H5N1	455
2004 Indonesia dengue epidemic	2004	Indonesia	Dengue fever	658
2004 Sudan ebola outbreak	2004	Sudan	Ebola	7
2005 dengue outbreak in Singapore	2005	Singapore	Dengue fever	27
2006 dengue outbreak in India	2006	India	Dengue fever	50+
Chikungunya outbreaks	2006	India	Chikungunya virus	Unknown (numerous widespread cases)
2006 dengue outbreak in Pakistan	2006	Pakistan	Dengue fever	50+
2006 Philippines dengue epidemic	2006	Philippines	Dengue fever	1,000

2006–07 East Africa Rift Valley fever outbreak	2006–2007	East Africa	Rift Valley fever	394
Mweka ebola epidemic	2007	Democratic Republic of the Congo	Ebola	187
2007 Puerto Rico, Dominican Republic, and Mexico dengue fever epidemic	2007	Puerto Rico, Dominican Republic, Mexico	Dengue fever	183
2007 Uganda ebola outbreak	2007	Uganda	Ebola	37
2008 Brazil dengue epidemic	2008	Brazil	Dengue fever	67
2008 Cambodia dengue epidemic	2008	Cambodia	Dengue fever	407
2008–2017 China hand, foot, and mouth disease epidemic	2008–2017	China	Hand, foot, and mouth disease	3,322+
2008 Philippines dengue epidemic	2008	Philippines	Dengue fever	172
2009 Bolivian dengue fever epidemic	2009	Bolivia	Dengue fever	18
2009 Gujarat hepatitis outbreak	2009	India	Hepatitis B	49
Queensland 2009 dengue outbreak	2009	Queensland, Australia	Dengue fever	1+ (503 cases)
Mumps outbreaks in the 2000s	2009	Worldwide	Mumps	Unknown

2009 swine flu pandemic	2009–2010	Worldwide	Influenza: A virus subtype H1N1	Lab confirmed deaths: 18,449 (reported to the WHO)
2010–2014 Democratic Republic of the Congo measles outbreak	2010–2014	Democratic Republic of the Congo	Measles	4,500+
2011 Vietnam hand, foot and mouth disease epidemic	2011	Vietnam	Hand, foot and mouth disease	170
2011 dengue outbreak in Pakistan	2011	Pakistan	Dengue fever	350+
2012 yellow fever outbreak in Darfur, Sudan	2012	Darfur, Sudan	Yellow fever	171
2012 Middle East respiratory syndrome coronavirus outbreak	2012–present	Worldwide	Middle East respiratory syndrome / MERS-CoV	935 (as of 4 July 2020[update])
2013 dengue outbreak in Singapore	2013	Singapore	Dengue fever	8
2013 Vietnam Measles outbreak	2013–2014	Vietnam	Measles	142
Western African Ebola virus epidemic	2013–2016	Worldwide, primarily concentrated in Guinea, Liberia, Sierra Leone	Ebola	11,323+
2013–14 chikungunya outbreak	2013–2015	Americas	Chikungunya	183

2013–19 Avian influenza epidemic	2013–2019	China	Influenza: A virus subtype H7N9	616
2014 Odisha jaundice outbreak	2014–2015	India	Primarily Hepatitis E, but also Hepatitis A	36
2015 Indian swine flu outbreak	2015	India	Influenza: A virus subtype H1N1	2,035
2015–16 Zika virus epidemic	2015–2016	Worldwide	Zika virus	53
2016 Angola and DR Congo yellow fever outbreak	2016	Angola and DR Congo	Yellow fever	498 (377 in Angola, 121 in Congo)
2017 dengue outbreak in Peshawar	2017	Peshawar, Pakistan	Dengue fever	69
2017 Gorakhpur Japanese encephalitis outbreak	2017	India	Japanese encephalitis	1,317
2017–18 United States flu season	2017–2018	United States	Seasonal Influenza	61,000 (46,000–95,000)[263]
2018 Nipah virus outbreak in Kerala	2018	India	Nipah virus infection	17
Kivu Ebola epidemic	2018–2020	Democratic Republic of the Congo and Uganda	Ebola	2,280
2019 measles outbreak in the	2019–2020	Democratic Republic of the	Measles	7,018+

Democratic Republic of the Congo		Congo		
2019–2020 New Zealand measles outbreak	2019–present	New Zealand	Measles	2
2019 Philippines measles outbreak	2019–present	Philippines	Measles	415
2019 Kuala Koh measles outbreak	2019	Kuala Koh, Malaysia	Measles	15
2019 Samoa measles outbreak	2019–present	Samoa	Measles	83
2019–20 dengue fever epidemic	2019–present	Asia-Pacific, Latin America	Dengue fever	3,930
COVID-19 pandemic	2019–present	Worldwide	COVID-19	1.6 million+ (as of Dec. 2020)
2019 Nigeria Lassa Fever epidemic	2019–present	Nigeria	Lassa fever	240 (as of 5 Dec. 2020)
2020 Democratic Republic of the Congo Ebola outbreak	2020–present	Democratic Republic of the Congo	Ebola	55
2020 novel bunyavirus outbreak	2020–present	China	Severe fever with thrombocytopenia syndrome	7 (as of Aug. 2020)
2020 Nigeria yellow fever epidemic	2020–present	Nigeria	Yellow Fever	172 (as of 3 December 2020)

Note: The data in the table is from Wikipedia (https://en.wikipedia.org/wiki/List_of_epidemics) for historic outbreaks and WHO website for modern outbreaks (https://www.who.int/emergencies/diseases/en/).

Table S2 Contribution of viral lysing of bacteria to DOC within different ecosystems

Ecosystem type		CRC ‰	TCOE soil: gC·kg^{-1} /water: mgC·L^{-1}	VLBC soil: mgC·kg^{-1} /water: μgC·L^{-1}	FVIC %	FMVL %	BCP soil: mgC·kg^{-1} /water: μgC·L^{-1}	BP 10^{9}cell·L^{-1}
Terrestrial	Wetland	0.8-4.4 (2.6)	218.7-345.8 [1]	273.5-968.4	—	27.0-54.9 [2]	1012.8-1764.0 [1]	—
	Cropland	1.3-4.5 (2.9)	13.4-14.3 [1]	18.7-60.4	4.2-11.4 [3]	7.9-23.5	237.6-256.8 [1]	—
	Pasture	6.6-10.8 (8.7)	40.1-50.0 [1]	328.7-431.7	23.0 [4]	56.8	578.4-759.6 [1]	—
	Tundra	2.9-22.2 (12.6)	189.3-316.8 [1]	927.1-4202.3	15.0-27.0 [5]	32.6-71.6	2844.0-5869.2 [1]	—
Limnetic zone	Lake	0.4-8.4 (4.4)	5.7 [6]	2.1-47.6	0.5-9.0 [7]	0.8-17.9	266.0	13.3 [8]
	River	0.7-61.5 (31.1)	3.2-9.9 [9]	6.7-196.8	—	2.5-74.0 [10]	266.0	13.3 [8]
Marine	Offshore	0.3-4.0 (2.2)	0.8	0.2-3.2	0.2-3.9	0.4-7.2	44.0	2.2

		[11]		[7]			[8]
Coastal	0.1~1.0 (0.6)	3.4 [12]	0.5~3.4	0.7~4.1 [7]	1.2~7.7	44.0	2.2 [8]

Note: CRC is the contribution rate of C by virus lysing of bacteria to ecosystem DOC, with the mean values in brackets; TCOE is the total DOC concentration of the ecosystem; VLBC is the C production produced by viral lysing of bacteria (soil: mg $C \cdot kg^{-1}$/water: μg $C \cdot L^{-1}$); FVIC is the frequency of visibly infected cells as seen under an electron microscope; FMVL is the fraction of mortality from viral lysis; BCP is bacterial C production; BP is bacterial production.

Table S3 Data sources of virus abundance shown in Figure 3

Marine				
Offshore (L^{-1})	Coastal (L^{-1})	Deep sea (>1000m) (L^{-1})	Sediment (gdw^{-1})	Source references
9.7×10^{6}	4.0×10^{7}	1.6×10^{6}	8.6×10^{9}	[8]
			1.3×10^{9}	[8]
			8.2×10^{7}	[13]
			5.8×10^{7}	[13]
			4.0×10^{8}	[13]
			3.6×10^{8}	[13]
			2.4×10^{9}	[13]
			2.0×10^{9}	[13]
			1.2×10^{9}	[13]
			1.1×10^{10}	[13]
			1.1×10^{9}	[13]
			8.0×10^{7}	[13]
			6.0×10^{7}	[13]
	8.4×10^{9}			[14]
	4.9×10^{7}			[15]
5.0×10^{9}	9.0×10^{9}			[16]
9.7×10^{9}	2.0×10^{9}			[16]
1.6×10^{10}	6.9×10^{10}			[16]
3.9×10^{9}				[16]
3.4×10^{9}		5.1×10^{8}		[17]
5.1×10^{9}		5.2×10^{8}		[17]
5.2×10^{9}		8.9×10^{8}		[17]
1.5×10^{10}	5.8×10^{9}			[18]
6.0×10^{7}	5.8×10^{9}			[18]
1.9×10^{9}	5.2×10^{10}			[18]
4.8×10^{9}	1.5×10^{11}			[18]
3.0×10^{6}	3.3×10^{10}			[18]
4.6×10^{11}	3.5×10^{10}			[18]
1.9×10^{9}	3.4×10^{10}			[18]
6.2×10^{8}	3.4×10^{10}			[18]
1.3×10^{9}	9.4×10^{9}			[18]
8.9×10^{9}	2.0×10^{10}			[18]
	1.6×10^{10}			[18]
	2.0×10^{9}			[18]
	1.5×10^{9}			[18]
	2.3×10^{9}			[18]
	4.4×10^{8}			[18]
	3.8×10^{10}			[18]

	1.8×10^{10}			18

Freshwater				
River (L^{-1})	River sediment (gdw^{-1})	Lake (L^{-1})	Lake sediment (gdw^{-1})	Source references
$5. 6\times10^{7}$		3.5×10^{7}	6.9×10^{9}	[8]
		7.5×10^{7}	1.1×10^{10}	[8]
			1.5×10^{9}	[13]
			6.7×10^{8}	[13]
1.4×10^{8}	6.8×10^{6}	5.4×10^{7}		[10]
7.4×10^{7}	4.2×10^{8}	1.6×10^{7}		[10]
1.5×10^{8}		8.6×10^{6}		[10]
6.9×10^{7}		5.0×10^{7}		[10]
2.1×10^{7}		1.1×10^{7}		[10]
4.6×10^{7}		2.6×10^{7}		[10]
1.5×10^{8}		8.5×10^{7}		[10]
1.9×10^{7}		3.7×10^{7}		[10]
4.0×10^{7}		6.4×10^{7}		[10]
4.8×10^{8}		6.5×10^{5}		[10]
1.1×10^{7}		1.7×10^{7}		[10]
2.5×10^{7}				[10]
7.8×10^{7}				[10]
3.4×10^{7}				[10]
8.9×10^{6}				[10]
8.8×10^{6}				[10]
5.0×10^{7}				[10]

Glacier			
Ice (L^{-1})	Water in cryoconite holes (L^{-1})	Sediment of cryoconite holes (g^{-1})	Source references
2.9×10^{2}	7.2×10^{2}		[19]
	6.8×10^{1}		[20]
		6.3×10^{8}	[21]
		7.5×10^{8}	[21]
		1.1×10^{9}	[21]

Note: Terrestrial ecosystem data all derived from Williamson et al. (2017) [22].

Table S4 Data sources of ecosystem C reserves and C exchange volume shown in Figure 4

Cause of death	Value	Value	Source references
C exchange	Primary producer respiration	45 GtC yr^{-1}	[23]
	Fossil fuel	6 GtC yr^{-1}	[23,24]
	Photosynthesis of terrestrial plants	120 GtC yr^{-1}	[23]
	Soil respiration	75 GtC yr^{-1}	[23]
	Photosynthesis of marine primary producers	93 GtC yr^{-1}	[25]
	Marine respiration	90 GtC yr^{-1}	[23]
	Release of organic matter via the viral shunt pathway	10 GtC day^{-1}	[26]
	Soil organic carbon (SOC) deposition	1 GtC yr^{-1}	[27]
	Marine particulate organic carbon (POC) deposition	3 GtC yr^{-1}	[25]
C storage	Terrestrial plants	560 GtC	[24]
	Soil DOC	2 GtC	[28]
	Oceanic primary producers	5 GtC	[25]
	Oceanic DOC	700 GtC	[25]
	Soil organic C pool	1550 GtC	[24]

	Marine organic C pool	2500 GtC	[29]
	Atmospheric C pool	805 GtC + 4 GtC yr^{-1}	[25]
	Fossil fuels	7000 GtC	[25]
Percentages of the steady-state model on the influence of viruses in marine C cycles	Primary producer photosynthesis	100%	[30]
	Entering DOC pool via viral-induced lysis	6%~26%	
	Primary producers – DOC	<10%	
	Primary producers – Viral shunt	2%~10%	
	Heterotrophic bacterial – Viral shunt	3%~15%	
	Consumers – Viral shunt	1%	
	Heterotrophic bacterial – Grazers	3%~9%	

Table S5 Illnesses and the number of annual deaths caused by viral infections in 2016, 2015, 2010 and 2000 (unit: year^{-1})

Cause of death	2016	2015	2010	2000	Mean
All causes	1,901,442,060	1,913,161,129	1,984,428,277	2,209,702,684	2,002,183,538
HIV/AIDS	55,922,099	58,950,489	83,319,436	86,989,523	71,295,387
Measles	7,935,125	10,973,429	13,876,495	57,429,582	22,553,658
Encephalitis	4,704,406	4,770,622	5,632,655	8,039,862	5,786,886
Hepatitis	7,185,133	7,333,652	7,829,938	8,719,188	7,766,978
Lower respiratory infections	129,208,762	130,969,710	152,621,131	204,732,197	154,382,950
Upper respiratory infections	230,354	215,094	297,627	388,889	282,991
Total	205,185,879	213,212,996	263,577,281	366,299,241	262,068,849
Viral proportion	0.108	0.111	0.133	0.166	0.129

Note: "Viral proportion" represents the percentage of deaths caused by virus infection in total deaths.

References

1 Xu, X. F., Thornton, P. E. & Post, W. M. A global analysis of soil microbial biomass carbon, nitrogen and phosphorus in terrestrial ecosystems. *Global Ecology and Biogeography* **22**, 737-749 (2013).

2 Li, X. L. *Bacteriophage diversity, abundence and its role in the production of disolved organic carbon in Napahai plateau wetland* doctor thesis, Kunming University Of Science And Technology, (2015).

3 Takahashi, R., Saka, N., Honjo, H., Asakawa, S. & Kimura, M. Comparison of the frequency of visibly infected bacterial cells between the soil and the floodwater in two Japanese rice fields. *Soil Science and Plant Nutrition* **59**, 331-336 (2013).

4 Bowatte, S., Newton, P. C. D., Takahashi, R. & Kimura, M. High frequency of virus-infected bacterial cells in a sheep grazed pasture soil in New Zealand. *Soil Biology and Biochemistry* **42**, 708-712 (2010).

5 Bellas, C. M. *et al.* Viral impacts on bacterial communities in Arctic cryoconite. *Environmental Research Letters* **8**, 045021 (2013).

6 Sobek, S., Tranvik, L. J., Prairie, Y. T., Kortelainen, P. & Cole, J. J. Patterns and regulation of dissolved organic carbon: An analysis of 7,500 widely distributed lakes. *Limnology and Oceanography* **52**, 1208-1219 (2007).

7 Weinbauer, M. G. Ecology of prokaryotic viruses. *Fems Microbiology Ecology* **28**, 127-181 (2004).

8 Parikka, K. J., Le Romancer, M., Wauters, N. & Jacquet, S. Deciphering the virus-to-prokaryote ratio (VPR): insights into virus–host relationships in a variety of ecosystems. *Biological Reviews* **92**, 1081-1100 (2016).

9 Dai, M., Yin, Z., Meng, F., Liu, Q. & Cai, W.-J. Spatial distribution of riverine DOC inputs to the ocean: an updated global synthesis. *Current Opinion in Environmental Sustainability* **4**, 170-178 (2012).

10 Peduzzi, P. Virus ecology of fluvial systems: a blank spot on the map? *Biological Reviews* **91**, 937-949 (2015).

11 Arıstegui, J. *et al.* Dissolved organic carbon support of respiration in the dark ocean. *Science* **298**, 1967-1967 (2002).

12 Barrón, C. & Duarte, C. M. Dissolved organic carbon pools and export from the coastal ocean. *Global Biogeochemical Cycles* **29**, 1725-1738 (2015).

13 Danovaro, R., Manini, E. & Dell'Anno, A. Higher abundance of bacteria than of viruses in deep Mediterranean sediments. *Applied and Environmental Microbiology* **68**, 1468-1472 (2002).

14 Finke, J. F. *Environmental and genomic insights into marine virus populations and communities*, University of British Columbia, (2017).

15 Hewson, I., Vargo, G. & Fuhrman, J. Bacterial diversity in shallow oligotrophic marine benthos and overlying waters: effects of virus infection, containment, and nutrient enrichment. *Microbial Ecology* **46**, 322-336 (2003).

16 Jover, L. F., Effler, T. C., Buchan, A., Wilhelm, S. W. & Weitz, J. S. The elemental composition of virus particles: implications for marine biogeochemical cycles. *Nature Reviews Microbiology* **12**, 519-528 (2014).

17 Lara, E. *et al.* Unveiling the role and life strategies of viruses from the surface to the dark ocean. *Science Advances* **3**, e1602565 (2017).
18 Proctor, L. M. Advances in the study of marine viruses. *Microscopy Research and Technique* **37**, 136-161 (1997).
19 Anesio, A. M., Mindl, B., Laybourn-Parry, J., Hodson, A. J. & Sattler, B. Viral dynamics in cryoconite holes on a high Arctic glacier (Svalbard). *Journal of Geophysical Research: Biogeosciences* **112** (2007).
20 Sawstrom, C., Mumford, P., Marshall, W., Hodson, A. & Laybourn-Parry, J. The microbial communities and primary productivity of cryoconite holes in an Arctic glacier (Svalbard 79 degrees N). *Polar Biology* **25**, 591-596, doi:10.1007/s00300-002-0388-5 (2002).
21 Bellas, C. M. *et al.* Viral impacts on bacterial communities in Arctic cryoconite. *Environmental Research Letters* **8**, doi:10.1088/1748-9326/8/4/045021 (2013).
22 Williamson, K. E., Fuhrmann, J. J., Wommack, K. E. & Radosevich, M. Viruses in soil ecosystems: an unknown quantity within an unexplored territory. *Annual Review of Virology* **4**, 201-219 (2017).
23 Schlesinger, W. H. & Andrews, J. A. Soil respiration and the global carbon cycle. *Biogeochemistry* **48**, 7-20 (2000).
24 Lal, R. Soil erosion and the global carbon budget. *Environment International* **29**, 437-450 (2003).
25 Suttle, C. A. Viruses in the sea. *Nature* **437**, 356-361 (2005).
26 Coutinho, F. H. *et al.* Marine viruses discovered via metagenomics shed light on viral strategies throughout the oceans. *Nature Communications* **8**, 1-12 (2017).
27 Lal, R. Soil carbon sequestration impacts on global climate change and food security. *science* **304**, 1623-1627 (2004).
28 Huang, Q. *et al.* Adsorption of Dissolved Organic Carbon (DOC) on Soil: A Review. *Soils* **47**, 446-452 (2015).
29 Zimov, S. A., Schuur, E. A. & Chapin III, F. S. Permafrost and the global carbon budget. *Science (Washington)* **312**, 1612-1613 (2006).
30 Wilhelm, S. W. & Suttle, C. A. Viruses and Nutrient Cycles in the Sea. *Bioscience*, 10 (1999).

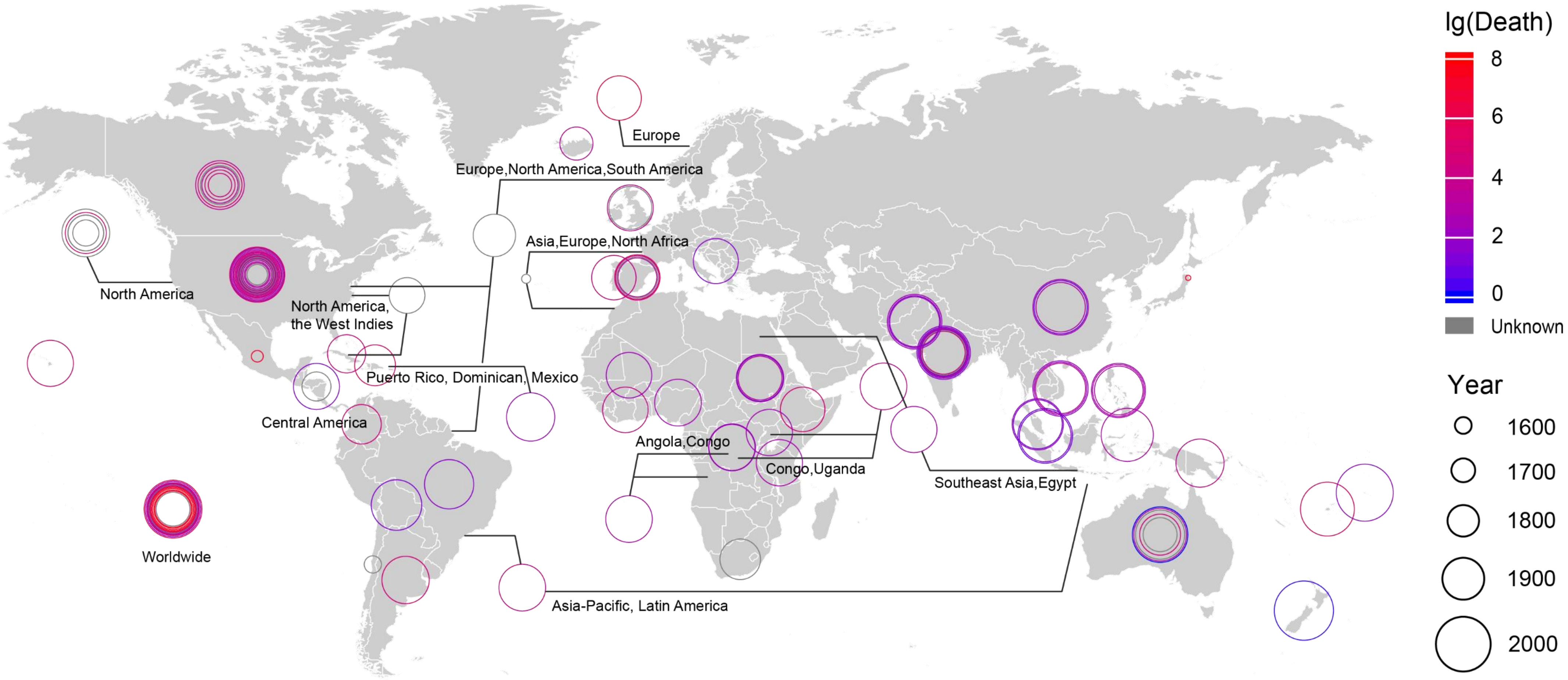

Figure S1 The outbreak time, location and death toll of all viral epidemics on record. See Table S1 for detailed information.

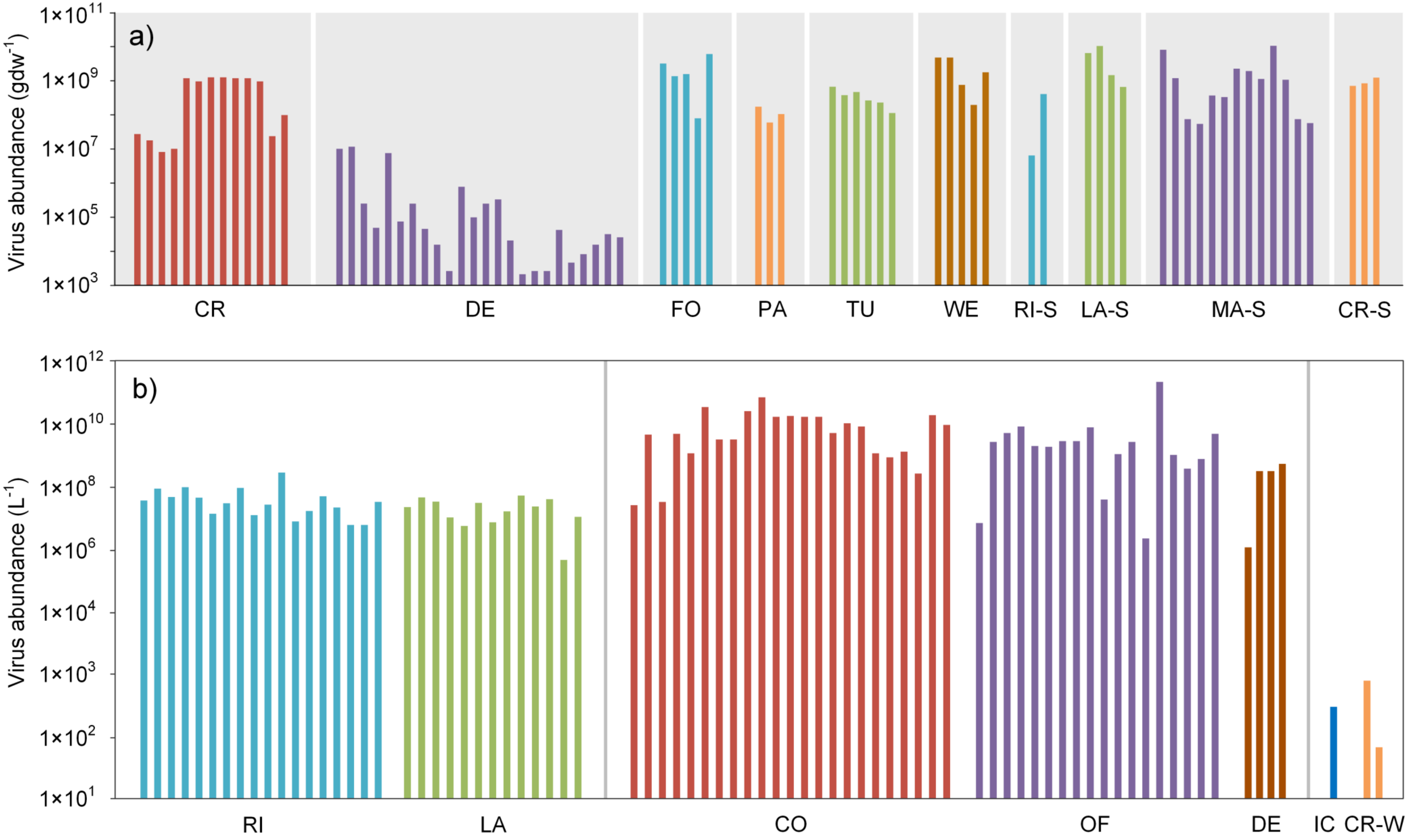

a)
Virus abundance (gdw-1)
1×10^11
1×10^9
1×10^7
1×10^5
1×10^3
CR
DE
FO
PA
TU
WE
RI-S
LA-S
MA-S
CR-S
b)
Virus abundance (L-1)
1×10^12
1×10^10
1×10^8
1×10^6
1×10^4
1×10^3
1×10^2
1×10^1
RI
LA
CO
OF
DE
IC
CR-W

Figure S2 Virus abundance within different ecosystems. CR: cropland; DE: Desert; FO: Forest; PA: Pasture; TU: Tundra; WE: Wetland; RI-S: River-Sediment; LA-S: Lake-Sediment; MA-S: Marine-Sediment; CR-S: Cryoconite holes-Sediment; RI: River; LA: Lake; CO: Coastal; OF: Offshore; DE: Deep sea; IC: Ice; CR-W: Cryoconite holes-Water. See Table S3 for data sources.
